# Integrative Medicine in Interventional Oncology: A Virtuous Alliance

**DOI:** 10.3390/medicina56010035

**Published:** 2020-01-17

**Authors:** François H. Cornelis, Milan Najdawi, Mohamed Ben Ammar, Maud Nouri-Neuville, Bénédicte Lombart, Jean-Pierre Lotz, Jacques Cadranel, Matthias Barral

**Affiliations:** 1 Department of Interventional Radiology and Oncology, Tenon Hospital, Sorbonne University, 4 rue de la Chine, 75020 Paris, France; milannajdawi@gmail.com (M.N.); mohamed.benammar@aphp.fr (M.B.A.); maud.nourineuville@aphp.fr (M.N.-N.);; 2 Saint Antoine Hospital, Sorbonne University, 184 rue du Faubourg Saint Antoine, 75012 Paris, France; benedicte.lombart@aphp.fr; 3 Department of Medical Oncology, Tenon Hospital, Sorbonne University, 4 rue de la Chine, 75020 Paris, France; jean-pierre.lotz@aphp.fr; 4 Department of Pneumology, Tenon Hospital, Sorbonne University, 4 rue de la Chine, 75020 Paris, France; jacques.cadranel@aphp.fr

**Keywords:** cancer, clinical hypnosis, in which cancer patients can receive integrative medicine, interventional oncology, virtual reality

## Abstract

This review aimed to identify the potential role of integrative medicine in interventional oncology. The music therapy; stress management techniques; guided imagery, including virtual reality; clinical hypnosis; and digital sedation may all be efficient on anxiety and pain during procedures performed in interventional oncology. Beyond pharmacological sedation, the implementation of integrative medicine to interventional oncology may, therefore, improve the support and care of cancer patients, which may further create a virtuous alliance.

## 1. Introduction

Interventional oncology (IO) offers minimally invasive diagnostic and therapeutic procedures to manage certain cancer-related problems [1,2]. These image-guided procedures include biopsies and treatments such as embolization, percutaneous ablation, or cementoplasty, allowing local control or symptom relief [2,3,4,5]. They are mainly performed under local anesthesia by interventional radiologists (IR) but they may cause anxiety and pain to patients [6,7,8,9]. Therefore, additional management should be implemented in concert to better take into account the patient′s well-being [9], including integrative medicine (IM).

By considering the whole person and their lifestyle, IM promotes the therapeutic relationship between practitioners and the patient [10]. IM techniques can be implemented relatively easily before, during, and after all IO procedures to manage anxiety and pain leading to improvement in both the quality of life and the well-being of cancer patients [6]. The objectives of this review are to describe the rationale of IM and to detail the techniques that can be used in IO. A MEDLINE/PubMed literature search was performed using keywords such as “interventional oncology” and “integrative medicine”. The articles published between January 2000 and December 2019 were selected based on their relevance, as well as cited references.

## 2. Definition of Integrative Medicine

The aim of Integrative medicine (IM) is to improve the patient′s health by combining some alternative medicine (AM) with traditional conventional medical therapies [11]. IM considers the patient′s unique conditions, needs, and circumstances [12]. Therefore, IM places the patient at the center of a personalized therapy strategy. IM differs from AM, which refers only to therapies used instead of conventional therapies [12]. Unlike AM, only therapies that have high-quality scientific evidence of safety and efficacy such as meditation, music therapy, and clinical hypnosis are combined with medical treatments in IM [11,13].

Due to the great needs of cancer patients, IM was thoroughly evaluated in this population [13]. To protect patients from potentially harmful treatments, and to manage symptoms and side effects before, during, or after treatment, some guidelines exist for the use of IM, such as in breast carcinoma [12,13]. A dedicated IM program helps to provide effective and appropriate care [14].

IM goes beyond conventional treatments to address all causes of discomfort, including anxiety and pain, both of which affect well-being [15]. Therefore, to address the full range of biological, behavioral, psychosocial, and environmental influences that can affect a person′s care, immediate needs and complex interactions between all influences are taken into account [16]. Everything that can influence health, well-being, and illness are considered, including body, mind, spirit, and community. Prevention or healthy behaviors are promoted. The development of skills that patients can use throughout their lives is encouraged. Less invasive procedures, which may include IO procedures, are preferred wherever possible and take full advantage of the effectiveness of techniques developed in IM [14].

## 3. Implementation in Interventional Oncology

The IR must provide each patient with adequate analgesia and sedation to manage anxiety and pain, and potentially amnesia, from the procedures they perform [8,9,17]. Besides minimizing the risk of complications, they must improve patient comfort before, during, and after the intervention. An uncomfortable and unfamiliar environment or the fear of loss of control may increase stress [9]. Although anesthesiologists are better trained to achieve these goals, they are not available to assist with all IO procedures. As sedation poses significant risks to patients [9], alternative techniques developed in IM can be used in combination with minimally invasive image-guided procedures developed in IO to alleviate anxiety and pain while ensuring better diagnosis and results.

### 3.1. Relational Commitment

As anxiety is often correlated with the patient’s relationship with professionals, the elimination of negative language from dialogue, replaced by positive or neutral language, may create self-confidence and relaxation [9,18,19]. As IM requires ongoing education and training to ensure patients receive multimodal treatment based on the unique needs of each patient, a dedicated consultation before any procedure can help relieve symptoms associated with cancer and also identify the IM technique that best suits the patient’s needs. Interactive wall projections in the IO waiting room can also be helpful. Having an IM service in a hospital system will certainly help to ensure coordinated patient care and to optimize the patient path to and from the operating rooms [14]. A specific data collection and analysis program must also be developed to obtain the mandatory high-quality scientific evidence of safety and efficacy, integrating new technologies such as artificial intelligence (AI). For example, to further strengthen the partnership between patients and practitioners as well as to assess the additional value of IM to IO, patients, family members, and staff could regularly use smartphone health apps to collect levels of self-reported well-being, pain, and anxiety, or satisfaction.

### 3.2. Naturopathic Medicine

Naturopathic medicine encourages the inherent process of self-healing [14]. Nutrition, herbal medicine, homeopathy, lifestyle counseling, and mind–body medicine are promoted [14]. While many of these techniques are beyond the scope of this review, nutrition may be interesting to evaluate in IO. For example, curcumin has been used for millennia because it improves immunity against cancer. Interestingly, experimental studies in mice have demonstrated significant inhibition of tumor growth after combining curcumin with percutaneous cryoablation [20]. These results are supported by several preclinical and clinical studies demonstrating that percutaneous ablations combined with appropriate immunomodulators can induce a therapeutically effective systemic anti-tumor immune response [21]. However, as some supplements or herbs can interfere with certain medications by altering their absorption, metabolism, or excretion, all treatment plans must be discussed to ensure that all practitioners are aware of this medication.

### 3.3. Music Therapy

Anxiety and pain can be mitigated by auditory inputs [22]. Music therapy has relaxing and distracting effects. Pain and auditory pathways have been suggested to inhibit each other [22]. Music therapy can be passive when the patient only listens to music, or active when the patient is creating live music. Significant improvement in anxiety, drowsiness, depression, nausea, fatigue, pain, and shortness of breath were demonstrated in a clinical study [23]. Music therapy decreases the need for sedation and pain relievers [22,24].

### 3.4. Meditation and Stress Management Techniques

Meditation, including yoga, qi gong, and tai chi chuan, aims to promote calm and concentration [12]. It allows participants to bring mental processes under greater voluntary control [25]. However, in addition to specific training, it requires a quiet location to limit distractions of concentration and attention. It also often needs a specific and comfortable posture that can be a challenge in the operating room.

Stress management techniques are very low-risk interventions inducing a parasympathetic response [14,26,27,28]. They are only contraindicated in patients with psychotic disorders. Progressive muscle relaxation, autogenic training, deep breathing, biofeedback, and guided imagery decrease anxiety [29]. These techniques must be taught beforehand to induce a relaxation response during interventions through stress management programs.

Among these techniques, guided imagery uses thoughts and imagination towards specific scenes to guide patients to achieve a specific objective [30]. Guided imagery reduces anxiety and pain, and can improve a person′s overall sense of well-being [31]. Face-to-face individual sessions are recommended to adapt the script to the patient′s life circumstances, although recordings are easier to implement and use. Virtual reality (VR) technology has been studied to facilitate access to this technique [32,33,34,35,36,37,38]. VR further isolates a patient from their direct environment by distracting their attention. Randomized studies have demonstrated an effect on the perception of anxiety and pain [39]. For example, VR reduces pain during burns or punctures [40].

### 3.5. Clinical Hypnosis

The principle of clinical hypnosis in IO is to create a modified state of consciousness of sensory elements. It is based on verbal suggestions made by a professional. The first objective is to relax the body and shift attention to a narrow range of objects or ideas. Secondly, the practitioner leads the patient to a dissociative state [41,42,43,44,45,46]. This trance state allows the patient to focus without distraction on specific feelings, thoughts, images, sensations, or behaviors [12].

Studies have shown that the effects of clinical hypnosis are similar to intravenous sedation [41], helping to reduce the need for medication during procedures [47,48,49] as well as costs [50]. Unfortunately, clinical hypnosis has had limited development in IO, which has been linked to professionals′ exposure to X-rays [51], as well as a lack of training and limited standardization of the technique [52,53,54].

### 3.6. Digital Sedation

**Figure 1:**
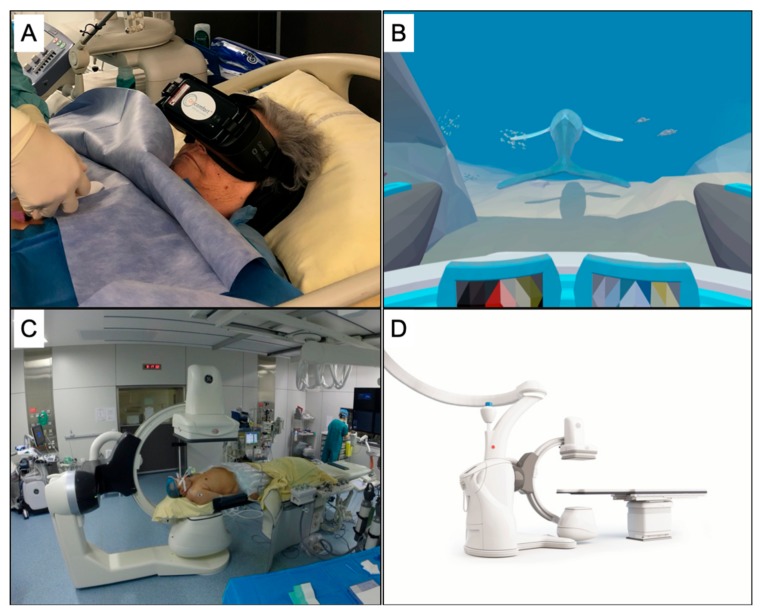
Integrative medicine in interventional oncology. (**A**) Digital sedation is provided by a virtual reality mask and headphones. (**B**) A screenshot of the digital sedation system. The patient follows a whale and has to adjust his breath according to the motion of the tail of the whale (courtesy of Oncomfort SA, Bruxelles, Belgium). (**C**) Interventional oncology operating rooms are often first designed to incorporate functions supporting highly advanced medical care, such as cone beam computed tomography, before considering patient well-being. (**D**) Future facilities using mobile robotics-assisted medical systems will allow to easily perform in-suite integrative medicine techniques and to improve the patient pathway to and from the operative rooms, limiting additional stress and anxiety (Discovery cone beam computed tomography system, courtesy of General Electric Healthcare, Buc, France).

Beyond using VR as a distraction tool and performing clinical hypnosis remotely using headphones, digital sedation (DS) has been developed [9]. To produce a dissociative state similar to clinical hypnosis without direct interaction with a professional, DS captures all of the patient’s attention by combining visual but also auditory sensations and verbal suggestions using a VR mask and headphones (Figure 1A) [55]. DS uses a multidimensional immersive VR technology, which is much more effective than traditional distraction methods like video games or movies [56,57]. Following a script, DS suggests different sensations that patients sometimes find it difficult to imagine through verbal suggestions (Figure 1B) [52,58,59].

Therefore, interesting standardized clinical applications can be developed for use during procedures performed in IO to limit anxiety and pain, similarly to clinical hypnosis or guided imagery, while limiting radiation exposure to staff, not only during the procedure but also after that [60]. In a study comparing DS with sedation in endoscopic urological surgeries [61], DS was more effective in terms of patient and anesthesiologist satisfaction. Although evidence of the effectiveness of DS in reducing anxiety and pain is accumulating [39], further evaluation is still necessary.

### 3.7. Toward Designing New Interventional Oncology Facilities

Although going beyond the scope of this review, architecture, art, and design can inspire well-being through the creation of a human-centered environment. Medical buildings or medical systems are often first designed to incorporate functions that physically support very advanced medical care, such as those developed in IO [62] (Figure 1C). However, they are rarely designed to create self-confidence and relaxation. Exploring the relationship between the architecture or design of healing facilities and a healing environment that promotes IM must be done in the future development of health care facilities. IO suites and medical systems, such as X-rays, computed tomography scan, or magnetic resonance imaging used to guide IO procedures, should be designed to easily perform IM techniques and to improve the patient′s path to and from operating rooms, limiting additional stress and anxiety [62] (Figure 1D). These health facilities must avoid noise, integrate indoor and outdoor spaces as well as the demands of daily life, and combine sophisticated technologies, such as VR, and traditional architectural techniques to remain sustainable and affordable, and to create a new ekistic reality fully integrated into its existing regulations and environmental and historical landmarks [63,64].

## 4. Conclusions

IM may be helpful to reduce anxiety and pain during IO procedures. New technologies such as VR and AI can be used to design wellness-focused digitization throughout the patient journey inside and outside of hospitals. As both IO and IM work together to improve well-being, the partnership between IO and IM may further create a virtuous alliance, allowing improved support and care of cancer patients throughout the disease.

## References

[1] Cornelis F.H., Lotz J.P. (2019). Interventional Oncology: A new pillar for a comprehensive cancer care. Presse Med..

[2] Cornelis F.H. (2017). The interventional oncologist: The fourth musketeer of cancer care. Diagn. Interv. Imaging.

[3] Kelekis A., Cornelis F.H., Tutton S., Filippiadis D. (2017). Metastatic Osseous Pain Control: Bone Ablation and Cementoplasty. Semin. Interv. Radiol..

[4] Vroomen L., Petre E., Cornelis F.H., Solomon S., Srimathveeravalli G. (2017). Irreversible electroporation and thermal ablation of tumors in the liver, lung, kidney and bone: What are the differences?. Diagn. Interv. Imaging.

[5] Filippiadis D., Tutton S., Kelekis A. (2017). Pain management: The rising role of interventional oncology. Diagn. Interv. Imaging.

[6] Mueller P.R., Biswal S., Halpern E.F., Kaufman J.A., Lee M.J. (2000). Interventional Radiologic Procedures: Patient Anxiety, Perception of Pain, Understanding of Procedure, and Satisfaction with Medication—A Prospective Study. Radiology.

[7] Sheta S.A. (2010). Procedural sedation analgesia. Saudi J. Anaesth..

[8] Cashman J.N., Ng L. (2017). The management of peri- and postprocedural pain in interventional radiology: A narrative review. Pain Manag..

[9] Cornelis F., Monard E., Moulin M.A., Vignaud E., Laveissiere F., Ben Ammar M., Nouri-Neuville M., Barral M., Lombart B. (2019). Sedation and analgesia in interventional radiology: Where do we stand, where are we heading and why does it matter?. Diagn. Interv. Imaging.

[10] Klionsky D.J., Abdelmohsen K., Abe A., Abedin M.J., Abeliovich H., Acevedo Arozena A., Adachi H., Adams C.M., Adams P.D., Adeli K. (2016). Guidelines for the Use and Interpretation of Assays for Monitoring Autophagy.

[11] Rosenthal D.S., Dean-Clower E. (2005). Integrative Medicine in Hematology/Oncology: Benefits, Ethical Considerations, and Controversies. Hematol. Am. Soc. Hematol. Educ. Program.

[12] Greenlee H., DuPont-Reyes M.J., Balneaves L.G., Carlson L.E., Cohen M.R., Deng G., Johnson J.A., Mumber M., Seely D., Zick S.M. (2017). Clinical practice guidelines on the evidence-based use of integrative therapies during and after breast cancer treatment. CA Cancer J. Clin..

[13] Lyman G.H., Greenlee H., Bohlke K., Bao T., DeMichele A.M., Deng G.E., Fouladbakhsh J.M., Gil B., Hershman D.L., Mansfield S. (2018). Integrative Therapies During and After Breast Cancer Treatment: ASCO Endorsement of the SIO Clinical Practice Guideline. J. Clin. Oncol..

[14] Armstrong K., Lanni T., Anderson M.M., Patricolo G.E. (2018). Integrative medicine and the oncology patient: Options and benefits. Support. Care Cancer.

[15] Hall M., Bifano S.M., Leibel L., Golding L.S., Tsai S.L. (2018). The Elephant in the Room: The Need for Increased Integrative Therapies in Conventional Medical Settings. Children.

[16] Knowles L.M., Skeath P., Jia M., Najafi B., Thayer J., Sternberg E.M. (2016). New and Future Directions in Integrative Medicine Research Methods with a Focus on Aging Populations: A Review. Gerontology.

[17] Martin M.L., Lennox P.H. (2003). Sedation and analgesia in the interventional radiology department. J. Vasc. Interv. Radiol..

[18] Schupp C.J., Berbaum K., Berbaum M., Lang E.V. (2005). Pain and Anxiety during Interventional Radiologic Procedures: Effect of Patients’ State Anxiety at Baseline and Modulation by Nonpharmacologic Analgesia Adjuncts. J. Vasc. Interv. Radiol..

[19] Lang E.V., Hamilton D. (1994). Anodyne imagery: An alternative to i.v. sedation in interventional radiology. AJR Am. J. Roentgenol..

[20] Chandra D., Jahangir A., Cornelis F., Rombauts K., Meheus L., Jorcyk C.L., Gravekamp C. (2016). Cryoablation and meriva have strong therapeutic effect on triple-negative breast cancer. Oncoimmunology.

[21] Takaki H., Cornelis F.H., Kako Y., Kobayashi K., Kamikonya N., Yamakado K. (2017). Thermal ablation and immunomodulation: From preclinical experiments to clinical trials. Diagn. Interv. Imaging.

[22] Lunde S.J., Vuust P., Garza-Villarreal E.A., Vase L. (2019). Music-induced analgesia: How does music relieve pain?. Pain.

[23] Lopez G., Christie A.J., Powers-James C., Bae M.S., Dibaj S.S., Gomez T., Williams J.L., Bruera E. (2019). The effects of inpatient music therapy on self-reported symptoms at an academic cancer center: A preliminary report. Support. Care Cancer.

[24] Koch M.E., Kain Z.N., Ayoub C., Rosenbaum S.H. (1998). The Sedative and Analgesic Sparing Effect of Music. Anesthesiology.

[25] Walsh R., Shapiro S.L. (2006). The meeting of meditative disciplines and western psychology: A mutually enriching dialogue. Am. Psychol..

[26] Eisenberg D.M., Kaptchuk T.J., Post D.E., Hrbek A.L., O’Connor B.B., Osypiuk K., Wayne P.M., Buring J.E., Levy N.B. (2016). Establishing an Integrative Medicine Program Within an Academic Health Center: Essential Considerations. Acad. Med..

[27] Raybin J.L., Barr E., Krajicek M., Jones J. (2019). How Does Creative Arts Therapy Reduce Distress for Children With Cancer? A Metasynthesis of Extant Qualitative Literature. J. Pediatr. Oncol. Nurs..

[28] Deng G. (2019). Integrative Medicine Therapies for Pain Management in Cancer Patients. Cancer J..

[29] Trijsburg R.W., Van Knippenberg F.C., Rijpma S.E. (1992). Effects of psychological treatment on cancer patients: A critical review. Psychosom. Med..

[30] Patricolo G.E., Lavoie A., Slavin B., Richards N.L., Jagow D., Armstrong K. (2017). Beneficial Effects of Guided Imagery or Clinical Massage on the Status of Patients in a Progressive Care Unit. Crit. Care Nurse.

[31] Richardson M.A., Sanders T., Palmer J.L., Greisinger A., Singletary S.E. (2000). Complementary/Alternative Medicine Use in a Comprehensive Cancer Center and the Implications for Oncology. J. Clin. Oncol..

[32] Li A., Montaño Z., Chen V.J., Gold J.I. (2011). Virtual reality and pain management: Current trends and future directions. Pain Manag..

[33] Zeng N., Pope Z., Lee J.E., Gao Z. (2018). Virtual Reality Exercise for Anxiety and Depression: A Preliminary Review of Current Research in an Emerging Field. J. Clin. Med..

[34] Furman E., Jasinevicius T.R., Bissada N.F., Victoroff K.Z., Skillicorn R., Buchner M. (2009). Virtual reality distraction for pain control during periodontal scaling and root planing procedures. J. Am. Dent. Assoc..

[35] Gold J.I., Kim S.H., Kant A.J., Joseph M.H., Rizzo A. (2006). Effectiveness of virtual reality for pediatric pain distraction during i.v. placement. Cyberpsychol. Behav..

[36] Gold J.I., Belmont K.A., Thomas D.A. (2007). The Neurobiology of Virtual Reality Pain Attenuation. Cyberpsychol. Behav..

[37] Morris L.D., Louw Q.A., Grimmer-Somers K., Grimmer K. (2009). The Effectiveness of Virtual Reality on Reducing Pain and Anxiety in Burn Injury Patients. Clin. J. Pain.

[38] Ryu J.H., Park J.W., Nahm F.S., Jeon Y.T., Oh A.Y., Lee H.J., Kim J.H., Han S.H. (2018). The Effect of Gamification through a Virtual Reality on Preoperative Anxiety in Pediatric Patients Undergoing General Anesthesia: A Prospective, Randomized, and Controlled Trial. J. Clin. Med..

[39] Mallari B., Spaeth E.K., Goh H., Boyd B.S. (2019). Virtual reality as an analgesic for acute and chronic pain in adults: A systematic review and meta-analysis. J. Pain Res..

[40] Chan E., Foster S., Sambell R., Leong P. (2018). Clinical efficacy of virtual reality for acute procedural pain management: A systematic review and meta-analysis. PLoS ONE.

[41] Vanhaudenhuyse A., Laureys S., Faymonville M.E. (2014). Neurophysiology of hypnosis. Neurophysiol. Clin. Neurophysiol..

[42] Faymonville M.E., Boly M., Laureys S. (2006). Functional neuroanatomy of the hypnotic state. J. Physiol..

[43] Laureys S., Faymonville M.E., Degueldre C., Fiore G.D., Damas P., Lambermont B., Janssens N., Aerts J., Franck G., Luxen A. (2000). Auditory processing in the vegetative state. Brain.

[44] Rainville P., Hofbauer R.K., Bushnell M.C., Duncan G.H., Price D.D. (2002). Hypnosis Modulates Activity in Brain Structures Involved in the Regulation of Consciousness. J. Cogn. Neurosci..

[45] Rainville P., Carrier B., Hofbauer R.K., Bushnell C.M., Duncan G.H. (1999). Dissociation of sensory and affective dimensions of pain using hypnotic modulation. Pain.

[46] Schnur J.B., Kafer I., Marcus C., Montgomery G.H. (2008). Hypnosis to manage distress related to medical procedures: A meta-analysis. Contemp. Hypn..

[47] Lang E.V., Benotsch E.G., Fick L.J., Lutgendorf S., Berbaum M.L., Berbaum K.S., Logan H., Spiegel D. (2000). Adjunctive non-pharmacological analgesia for invasive medical procedures: A randomised trial. Lancet.

[48] Lang E.V., Berbaum K.S. (1997). Educating interventional radiology personnel in nonpharmacologic analgesia: Effect on patients’ pain perception. Acad. Radiol..

[49] Tefikow S., Barth J., Maichrowitz S., Beelmann A., Strauss B., Rosendahl J. (2013). Efficacy of hypnosis in adults undergoing surgery or medical procedures: A meta-analysis of randomized controlled trials. Clin. Psychol. Rev..

[50] Lang E.V., Rosen M.P. (2002). Cost analysis of adjunct hypnosis with sedation during outpatient interventional radiologic procedures. Radiology.

[51] Bartal G., Vano E., Paulo G., Miller D.L. (2014). Management of patient and staff radiation dose in interventional radiology: Current concepts. Cardiovasc. Interv. Radiol..

[52] Askay S.W., Patterson D.R., Sharar S.R. (2009). Virtual Reality Hypnosis. Contemp. Hypn..

[53] Hilgard E.R. (1975). The alleviation of pain by hypnosis. Pain.

[54] Morgan A.H., Hilgard J.R. (1978). The Stanford Hypnotic Clinical Scale for Adults. Am. J. Clin. Hypn..

[55] Hoffman H.G., Sharar S.R., Coda B., Everett J.J., Ciol M., Richards T., Patterson D.R. (2004). Manipulating presence influences the magnitude of virtual reality analgesia. Pain.

[56] Hoffman H.G., Seibel E.J., Richards T.L., Furness T.A., Patterson D.R., Sharar S.R. (2006). Virtual Reality Helmet Display Quality Influences the Magnitude of Virtual Reality Analgesia. J. Pain.

[57] Hoffman H.G., Richards T.L., Bills A.R., Van Oostrom T., Magula J., Seibel E.J., Sharar S.R. (2006). Using FMRI to study the neural correlates of virtual reality analgesia. CNS Spectr..

[58] Patterson D.R., Wiechman S.A., Jensen M., Sharar S.R. (2006). Hypnosis Delivered Through Immersive Virtual Reality for Burn Pain: A Clinical Case Series. Int. J. Clin. Exp. Hypn..

[59] Patterson D.R., Jensen M.P., Wiechman S.A., Sharar S.R. (2010). Virtual reality hypnosis for pain associated with recovery from physical trauma. Int. J. Clin. Exp. Hypn..

[60] Hoffman H.G., Richards T., Coda B., Richards A., Sharar S.R. (2003). The Illusion of Presence in Immersive Virtual Reality during an fMRI Brain Scan. Cyberpsychol. Behav..

[61] Moon J.Y., Shin J., Chung J., Ji S.H., Ro S., Kim W.H. (2018). Virtual Reality Distraction during Endoscopic Urologic Surgery under Spinal Anesthesia: A Randomized Controlled Trial. J. Clin. Med..

[62] Frasca-Beaulieu K. (1999). Interior design for ambulatory care facilities: How to reduce stress and anxiety in patients and families. J. Ambul. Care Manag..

[63] Schuster R.J., Weber M.L. (2003). Noise in the Ambulatory Health Care Setting. J. Ambul. Care Manag..

[64] Craw J.B. (1989). Environmental impact-design with care. Coll. Rev..

